# A pilot study of the effects of faculty status for medical librarians in the United States

**DOI:** 10.5195/jmla.2021.1138

**Published:** 2021-10-01

**Authors:** Sa'ad Laws

**Affiliations:** 1 sal2018@qatar-med.cornell.edu, Research and Education Librarian, Weill Cornell Medicine – Qatar, Education City, Qatar

**Keywords:** faculty status, medical librarians, faculty perceptions

## Abstract

**Objectives:**

Within many institutions, there are debates over whether medical librarians should be classified as faculty or professional staff, a distinction that may have considerable effect on the perception of librarians within their local institutions. This study is a pilot exploration of how faculty status may affect the professional experiences of academic medical librarians within their local institutions.

**Methods:**

Surveys were sent to 209 medical librarians listed as having some instructional function at Liaison Committee on Medical Education (LCME) accredited medical institutions in the United States. Survey responses were captured using Qualtrics survey tool and analyzed for frequencies and associations using SPSS version 27.

**Results:**

Sixty-four medical librarians at academic medical institutions completed the survey developed for this study. Of the respondents, 60.9% indicated that librarians at their institution have faculty status, while 71.9% believe that librarians at their institution should have faculty status. Ninety percent of librarians with faculty status reported that they are expected to generate scholarly materials, compared to 28% of those without faculty status.

**Conclusions:**

Many medical libraries offer faculty status to librarians. While many medical librarians are active in instruction, research, and other activities normally associated with faculty status, it is not clear if faculty status impacts how librarians are perceived by other health care workers within their institutions.

## INTRODUCTION

Many academic librarians within college and university libraries have held faculty appointments for decades. While the benefits of faculty status can often vary greatly for librarians, one major advantage is the potential for faculty status to elevate the perception of librarians within an institution, placing them on an even field with full-time teaching and research faculty. Faculty status is also viewed as a means of encouraging librarians to engage in research [1, 2]. Yet, despite these longstanding appointments and perceived benefits, the library literature shows disagreement regarding whether faculty appointments are appropriate for librarians. Some common arguments in this debate center on the perceived limitations of librarians' education, research training, and disciplinary expertise, which are viewed as essential for faculty status; others have suggested that faculty status can even detract and distract from the core service missions of librarianship [3–6].

While discussions over the appropriateness of faculty status for librarians have unfolded, the literature suggests that librarians increasingly have taken actives roles in both instruction and research, two of the main articulations of faculty status. Several studies have noted that many librarians have expanded their roles as instructors in both extent and depth, with librarians both leading instructional initiatives as well as responding to faculty and student-initiated requests [7–9]. This expanded teaching role has led to a general trend toward greater librarian integration within the curriculum through incorporation into curricular committees at the institutional level [7, 10, 11]. As a result, academic medical librarians now often find themselves working with teaching and clinical faculty as peers, engaging in many of the same teaching and learning activities as full-time faculty members. In addition to instruction, many librarians have developed, increased, or expanded their research skills and works. Several studies have examined how librarians have made an impact as researchers and the effect that this has had on them as professionals [12–15]. An interesting example of the increasing capacity of librarians for research is librarian participation within interprofessional research teams in a myriad of capacities [16–19].

To further understand how faculty status affects medical librarians, this exploratory study seeks to understand how medical librarians view and understand faculty status, focusing on how faculty status impacts their daily activities and how they are perceived by their colleagues throughout their institutions. To answer questions related to the role and identity of medical librarians, this study explores the following questions:

- At what rate do medical librarians have faculty status of any type?
- Does faculty status affect the instructional activities and roles of medical librarians?
- Does faculty status affect the research capabilities and expectations for medical librarians?

For clarity, this article uses the term of *academic librarians* to refer to any librarian that works in a college and university setting; *medical librarians* refers to librarians working at academic institutions that offer an undergraduate medical degree, such as those defined by Liaison Committee on Medical Education (LCME) in the United States and Canada [20]. Additionally, *academic faculty* or *teaching faculty* will represent any faculty member at an academic institution who bears some instructional responsibilities as part of their professional duties. Furthermore, *medical faculty* will refer to faculty that are employed, at least in part, by an academic medical institution and have some of their professional duties based in instructing undergraduate medical students.

## METHODS

This study is a cross-sectional, survey-based project. The survey used was based on the work of Galbraith et al. as well as the work of Ivey (see Supplement 1) [21, 22]. The survey used in this study is comprised of fourteen Likert, Yes/No, and multiple-choice questions. Questions were separated into “About you,” Instruction, and Research sections. Prior to data collection, the survey was reviewed by three medical librarians at the author's institution. Using this feedback, adjustments were made to the survey prior to execution. Potential participants were selected utilizing convenience sampling by reviewing freely available personnel data available on websites of the 155 LCME academic medical institutions in the United States. The author examined the library departmental websites of each institution for contact information of librarians that indicated some level of instructional duties. As such, catalogers, archivists, and directors were generally excluded. The justification for this exclusion was to survey medical librarians that would have significant experiences in both instruction and research. Faculty status was not part of the inclusion criteria for solicited participants and was not known to the researcher at the time of requesting participation. Surveys for this study were created using Qualtrics survey tool and sent to 204 medical librarians between March and May of 2020. The author conducted data analysis using SPSS version 27 and consisted of descriptive frequencies and association analysis. Where relevant, testing for significance of association utilized Fisher's exact test (noted with an * in the text), due to the small sample size. Prior to data collection, the study was reviewed and approved by the author's institutional review board.

## RESULTS

A total of 64 respondents completed the librarian-based survey, resulting in a 31.3% response rate. Most respondents (54.7%, n=35) had been working in the position of medical librarian for over six years and the majority (56.3%, n=36) had also been at their current position for over six years. The majority of respondents (70.3%, n=45) had an MLIS degree alone (or equivalent) as their highest level of education. Of the respondents, 26.6% (n=17) reported holding an additional master's degree beyond their MLIS; 3.1% (n=2) reported holding a PhD.

When respondents were asked about the faculty status of librarians at their institution, 60.9% (n=39) indicated that librarians at their institution hold faculty status. When asked if librarians at their institution should have faculty status, 71.9% (n=46) respondents said that librarians should have faculty status, while 14.1% (n=9) did not know if librarians should have faculty status. When comparing if librarians should hold faculty status between respondents with and without faculty status at their institutions, there was a significant association (Fisher's exact=11.616, *p*=0.003*), with 87.2% (n=34) of faculty status respondents indicating that librarians at their institution should have faculty status, while only 48% (n=12) of nonfaculty respondents indicating the same, and 24% (n=6) of nonfaculty respondents indicating that they did not know if librarians at their institution should have faculty status.

**Figure 1:**
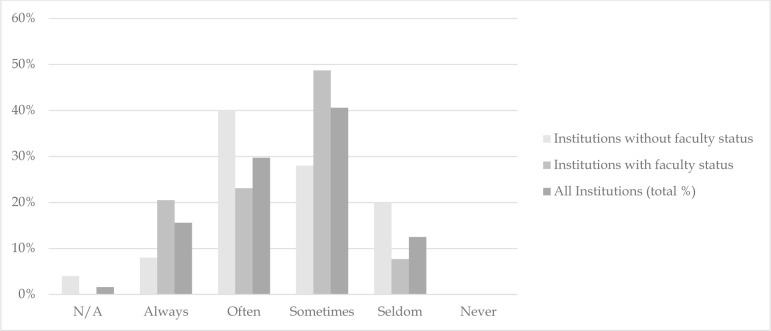
Librarians at my institution participate in the instruction of medical students

Respondents reported a range of involvement in instructional activities at their institutions, with 40.6% (n=26) indicating that librarians at their institutions “sometimes” participated in instruction and 29.7% (n=19) indicating that they “often” participated. When it came to faculty-librarian collaborations on course design and curricula, 43.8% (n=28) and 18.8% (n=12) of respondents noted that they were asked to participate “sometimes” and “often,” respectively. Conversely, 26.6% (n=17) and 6.3% (n=4) of respondents said they were “seldom” or “never” asked to participate. There was no significant association between faculty status and librarian involvement in instruction. Approximately the same level of “always” and “often” responses were reported by respondents who held faculty status and those who did not hold faculty status (Figure 1).

**Figure 2:**
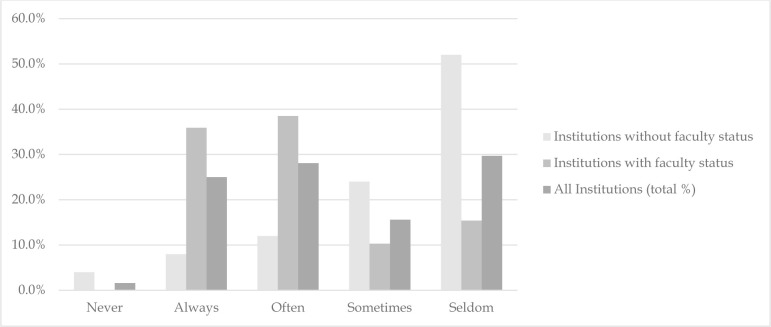
Librarians at my institution are active in conducting scholarly research

Respondents were asked several questions about the research activities of medical librarians at their institutions and their perceptions of those activities. When asked if medical librarians at their institutions were expected to produce scholarly materials as part of their job function, 65.6% (n=42) indicated yes, 31.3% (n=20) indicated no, and 1.6% (n=1) indicated that they did not know. When the above question was compared between those at institutions where respondents have faculty status and those without, there was a clear indication that faculty status can be associated with a greater expectation for scholarly activity. Of respondents from institutions with faculty status, 89.7% (n=35) indicated that librarians at those institutions are expected to produce scholarly materials, while only 28% (n=7) of respondents from institutions without faculty status answered similarly (Fisher's exact= 26.091, *p*=<0.001). Furthermore, when respondents were asked if librarians at their institutions were active in conducting scholarly research, there was a clear indication that respondents from institutions that offer faculty status were move active in scholarly activity, while those without faculty status were decidedly less active (Figure 2).

Additionally, respondents in this study were asked if medical librarians at their institutions possess the skills and knowledge to conduct scholarly research. Respondents offered a generally optimistic outlook, with 75% (n=48) of respondents indicating that they believed that librarians at their institutions have the required skills and knowledge for research activities. When this question was adjusted for institutions with and without faculty status, 24% (n=6) of nonfaculty respondents indicated that they did not know if medical librarians at their institute have the skills and knowledge to conduct research.

When asked about the primary purpose of medical librarians in their institution, there were no substantial differences between librarians with or without faculty. Of note was that both faculty and nonfaculty status librarians overwhelmingly selected research support as the primary function of medical librarians at their institution (all respondents: 46.9%, n=30). For respondents in this survey, functions like evidence-based medicine support (12.5%, n=8), instruction (12.5%, n=8), and other (18.8%, n=12) were reported as a primary function much less frequently.

There was no statistical association between faculty status and how respondents perceived the value of their research for the institution, specifically in comparison to medical faculty. Only 21.9% (n=14) of respondents either “always” or “often” saw the research products of librarians as on par with medical faculty. This is compared with 43.8% (n=28) of respondents that “never” or “seldom” saw their work as equal to medical faculty. In spite of these low appraisals of librarian-produced research, respondents generally indicated that faculty status could be beneficial when managing relationships with medical faculty; 76.9% (n=30) of respondents at institutions with faculty status indicated that faculty status does or could improve medical faculty perceptions of librarians; 48% (n=12) of respondents at institutions without faculty status indicated similarly. Of note, 40% (n=10) of librarians at institutions without faculty status indicated that they did not know if faculty status would improve medical faculty perceptions of librarians

## DISCUSSION

This exploratory study offers insights into how medical librarians view faculty status and how faculty status may be correlated with the types of activities librarians are expected to engage in at their institutions. Of the respondents, 60.9% (n=39) reported holding faculty status; this aligns with the findings of Bolin, who reported that 57% of Association of Research Libraries librarians had some form of faculty status [23]. Furthermore, a more recent study conducted by Walters found that 57% of academic librarians at nationally ranked US universities claim faculty status [24]; these data suggest that medical librarians hold faculty status at very similar rates as librarians at academic research libraries.

The research activities reported by respondents in this study are worth noting, as these data suggest a correlation between faculty status and the institutional expectations and perception for conducting scholarly research. These data indicate that medical librarians without faculty status were not as likely to be evaluated for conducting scholarly works, nor as active in conducting scholarly works. While this may seem like an obvious outcome, it suggests that certain evaluative measures, such as scholarly activity, may be more germane to medical librarians that hold faculty status than for those who do not. Studies have also shown that many librarians describe themselves as lacking in research skills or lacking in institutional support to conduct research [12, 25]. This study found that a significant portion of respondents at institutions with faculty status felt that librarians at their institution have the skills and knowledge to conduct research (84.6%, n=33). Given that many librarians indicate that they enter the field lacking in this area, this could suggest that faculty status was either a driving force for self-development in this area or that institutions that have faculty status offer greater support for research training. Future studies may wish to examine how faculty status impacts the professional development priorities of early-career librarians and also whether there are any differences in the quantity or quality of research production by librarians with and without faculty status.

Finally, it is of note that there was not a significant difference between faculty and nonfaculty librarians regarding instruction of medical students. Since a large portion of library-led instructional programming is provided at the request of medical faculty, this may indicate that medical faculty do not consider faculty status of a librarian important when requesting instructional assistance from medical librarians; the domain expertise and instructional skills of the librarian justify their invitation to the classroom rather than their employment classification. While there does not appear to be any study that has examined how teaching faculty differentiate instructional assistance based on librarian faculty status, studies have indicated varied levels of hesitancy by teaching faculty to engage with librarians for instruction for reasons other than faculty status [8, 26]. Despite this, it is of note that a larger percentage of librarians at institutions with faculty status indicated that faculty status was beneficial in their relationships with medical faculty. While additional research is needed in this area, it could be an indication that, while faculty status of librarians does not increase instructional opportunities, it does improve the ones that exist in terms of quality, depth, or significance [27].

Finally, the plurality of both faculty and nonfaculty status librarian respondents indicated that their primary function within their institution was to offer research support. Research support, unlike developing curricular-integrated instruction or conducting original research, may be regarded as a traditional service function of librarian. This may suggest that many respondents strongly identify with traditional, public service notions of librarianship, which may be viewed as less deserving of faculty status. Additional studies that include librarians that specialize in other functions of academic medical libraries (e.g., metadata librarianship or systems librarianship) could be useful in further exploring this finding.

## LIMITATIONS AND FUTURE RESEARCH

The current study is limited in the approach that was used to identify and solicit participants. While this method of convenience sampling is typically germane to pilot or exploratory studies such as this one, there is a possibility of introducing bias as a result. It will be important, based on the finding in this study, to conduct further research on the faculty status of medical librarians in a deeper fashion and with a larger sample. Future studies should also employ more complex data capture instruments that this study was not designed to capture, so that these studies can explore some of the complex and varied intersubjective relationships that exist for medical librarians in academic medical institutes. For example, this study indicates that medical librarians increasingly possess and appreciate faculty status; however, it will be important to question the nature of librarian faculty status. Is this status nominal or similar with other medical and nonmedical faculty in these institutions? As mentioned previously, it would be beneficial to know more about the institutional impact that medical librarian–based research has, particularly with clinical faculty and institutional administrators. Additionally, further studies could gather richer data on the specific instructional roles medical librarians hold and explore how these roles correlate based on their faculty status.

## Data Availability

The data used in deriving results for this research project are available in the OSF repository at https://osf.io/cn35y/?view_only=bc4fc0934ab84dad975a32066aaf7716.

## SUPPLEMENTAL FILES

**Supplement 1.** Medical librarian survey
