# Supplementary material for: A pilot study of the effects of faculty status for medical librarians in the United States
Source: J Med Libr Assoc. 2021 Oct 1;109(4):618–23. doi: 10.5195/jmla.2021.1138 (PMC8608164; doi:10.5195/jmla.2021.1138)
Supplement: Supplementary file 1 — Supplement 1. Medical librarian survey [file jmla-109-4-618-s01.pdf]

Consent

Version 3: January 26, 2020

An examination of medical faculty and librarians’ perceptions of the function and role of librarians within the academic medical institution

Informed Consent Statement

This research is being conducted to examine the following:

1. How do medical faculty perceive librarians’ standing and role within the institution?
2. How do librarians perceive their own role and standing within the institution?
3. What factors affect medical faculty perceptions of librarians?
4. How do librarians perceive medical faculty as partners in instruction and research?

You are being requested to participate in this survey because you are librarian within an academic medical institution located in the United States.

Your participation in this research project is completely voluntary and you can withdraw any time by not submitting the survey without penalty. The research team expects your participation to last approximately 3-5 minutes. It is estimated that a maximum of 500 librarian participants will take part in this study.

If you agree to be in this study, you will be asked to complete an online survey. You will be asked about your perception of medical librarians at your institution and their role and function within the institute. As a second wing of this research project, medical faculty are also being recruited to take a similar survey. When completed, this project will compare and contrast the findings from both surveys.

You are free to the study Principle Investigator about your involvement in this research at any time.

Inner: 819 x 599
Outer: 819 x 650

- Your questions, concerns, or complaints are not being answered by the research team.
- You cannot reach the research team.
- You want to talk to someone besides the research team.
- You have questions about your rights as a research subject.
- You want to get information or provide input about this research.

I hereby grant consent to my participation in the above research study

Yes

No

About You

How long have you been at your current institution?

Less than a year

1-2 Years

3 -4 Years

5 Years or more

How long, in total, have you been employed at any medical library

Less than a year

1-2 Years

3-4 Years

5-6 Years

7 Years or more

Do any librarians at your institution have faculty status?

Yes

No

I don't know

Inner: 819 x 599
Outer: 819 x 650

Faculty status does or could improve medical faculty's perception of librarians at my institution.

Yes

No

I don't know

What is your highest level of education?

Bachelor's

MLIS or similar

Other Masters

MLIS and Additional Master's

Ph.D.

Instruction

Librarians at my institution participate in the instruction of medical students?

Never	Seldom	Sometimes	Often	Always

Medical faculty value the instruction that librarians at my institution provide in support of their classes.

Never	Seldom	Sometimes	Often	Always

Librarians are asked by medical faculty at my institution to collaborate in designing and developing curricula for their courses.

Never	Seldom	Sometimes	Often	Always

The primary purpose of the librarians in my institution is: (choose one)

Collection Development / order books

Instruction

Research Support

Check out materials

Staffing library service desks

Evidence Based Medicine support

Systematic review support

I don't know

Other (Please specify)

Inner: 819 x 599
Outer: 819 x 650

I believe librarians at my institution should have faculty status.

Yes

No

I don't know

Research

Librarians at my institution are expected to produce scholarly material as part of their job duties.

Yes

No

I don't know

Librarians at my institution are active in conducting scholarly research.

Never	Seldom	Sometimes	Often	Always

Research produced by librarians at my institution is seen as equal value to the institution as research of medical faculty.

Never	Seldom	Sometimes	Often	Always

Librarians at my institution have the skills and knowledge to conduct scholarly research.

Yes

No

I don't know

Powered by Qualtrics

Inner: 819 x 599
Outer: 819 x 650
